# The magnitude of airway remodeling is not altered by distinct allergic inflammatory responses in BALB/c *versus* C57BL/6 mice but matrix composition differs

**DOI:** 10.1111/imcb.12448

**Published:** 2021-03-19

**Authors:** James E Parkinson, Stella Pearson, Dominik Rückerl, Judith E Allen, Tara E Sutherland

**Affiliations:** 1 Lydia Becker Institute of Immunology and Inflammation, Faculty of Biology, Medicine and Health, Manchester Academic Health Science Centre, University of Manchester, Manchester, UK; 2 Wellcome Centre for Cell-Matrix Research, Faculty of Biology, Medicine and Health, Manchester Academic Health Science Centre, University of Manchester, Manchester, UK

**Keywords:** airway remodeling, allergic airway inflammation, collagen, extracellular matrix, mouse strain differences, type 17 cytokines, type 2 cytokines

## Abstract

Allergic airway inflammation is heterogeneous with variability in immune phenotypes observed across asthmatic patients. Inflammation has been thought to directly contribute to airway remodeling in asthma, but clinical data suggest that neutralizing type 2 cytokines does not necessarily alter disease pathogenesis. Here, we utilized C57BL/6 and BALB/c mice to investigate the development of allergic airway inflammation and remodeling. Exposure to an allergen cocktail for up to 8 weeks led to type 2 and type 17 inflammation, characterized by airway eosinophilia and neutrophilia and increased expression of chitinase-like proteins in both C57BL/6 and BALB/c mice. However, BALB/c mice developed much greater inflammatory responses than C57BL/6 mice, effects possibly explained by a failure to induce pathways that regulate and maintain T-cell activation in C57BL/6 mice, as shown by whole lung RNA transcript analysis. Allergen administration resulted in a similar degree of airway remodeling between mouse strains but with differences in collagen subtype composition. Increased collagen III was observed around the airways of C57BL/6 but not BALB/c mice while allergen-induced loss of basement membrane collagen IV was only observed in BALB/c mice. This study highlights a model of type 2/type 17 airway inflammation in mice whereby development of airway remodeling can occur in both BALB/c and C57BL/6 mice despite differences in immune response dynamics between strains. Importantly, compositional changes in the extracellular matrix between genetic strains of mice may help us better understand the relationships between lung function, remodeling and airway inflammation.

## Introduction

Asthma is a global health problem with increasing prevalence, currently affecting over 300 million people.^1^ Of note, the term “asthma” encompasses a range of disease phenotypes. Progress in understanding heterogeneity of airway inflammation has led to defined asthma endotypes^2,3^ often characterized by the presence or absence of type 2 eosinophilic inflammation and/or type 17 neutrophilic inflammation.^4^ A clarified definition of such inflammatory phenotypes in asthma has led to development of innovative therapies directed at modulating specific inflammatory pathways, in particular type 2 inflammation.^5^ While the use of biologicals targeting immunoglobulin E or either type 2 cytokines interleukin (IL)-4, IL-5, IL-13 or their cognate receptors (IL-4Rα, IL-5R, IL-13Rα1) has been effective at reducing disease exacerbations in allergic asthmatics, these therapies are often insufficient to improve underlying disease pathogenesis.^6–8^ Furthermore, clinical trials with antibodies targeting IL-17 signaling have shown no benefit,^9^ despite a strong association of severe asthma with type 17 neutrophilic inflammation.^10^ To achieve progress in treating asthma we need a more comprehensive understanding of its pathology beyond viewing inflammation as the main instigator of disease.

Along with airway inflammation, asthma is characterized by airway hyperresponsiveness (AHR) and airway remodeling, a process of changes to the composition, content and organization of cells and extracellular matrix in the lung. Although tissue remodeling is a critical process during development and tissue repair,^11^ it is also a pathogenic response in diseases such as asthma, and undoubtedly impacts on lung function.^12^ Comprehensive research using a combination of mouse models and human studies typically attributes the development of remodeling to chronic airway inflammation.^13–17^ However, this view conflicts with emerging data that show remodeling can occur as a primary event prior to inflammation.^18–20^ Glucocorticoid steroids can generally improve lung function but do not alter airway remodeling.^21^ Alternatively, drugs that successfully target specific inflammatory pathways fail to improve lung function,^7,8^ presumably because they do not affect airway remodeling. Overall, the links between inflammation, remodeling and lung function are still unclear and thus warrant investigation. Airway remodeling is a complex disease process, difficult to study in patients especially in the context of understanding multiple components that may influence development of individual remodeling processes over time. Therefore, there is a crucial need for animal models that reflect asthma disease processes with a focus on studying the development of stable and irreversible airway remodeling.

Genetic differences between inbred mouse strains are well known to strongly affect both airway inflammation^22–25^ and AHR.^26^ For instance, C57BL/6 mice are known to have a high airway resistance in response to methacholine challenge independent of allergic inflammation,^22^ whereas BALB/c mice generally exhibit greater airway reactivity in response to allergens.^26^ Therefore, studies examining airway pathology in different mouse strains can provide a basis to explore the relationships between immune cell dynamics in relation to changes in airway remodeling and lung function. In this study we utilized a model of chronic allergic airway inflammation that shares features of disease common to severe asthma in people, including mixed type 2 and type 17 airway inflammation, steroid-resistant neutrophilia and AHR independent of type 2 cytokines,^27,28^ to investigate inflammation and remodeling parameters. Together, our results highlight mouse strain-dependent differences in type 2 and type 17 inflammation that do not seem to alter the development of remodeling but may impact on deposition of specific collagen subtypes.

## Results

### Chronic Allergen-Induced Immune Response Dynamics Differ Between C57BL/6 and BALB/C Mouse Strains

**Figure 1:**
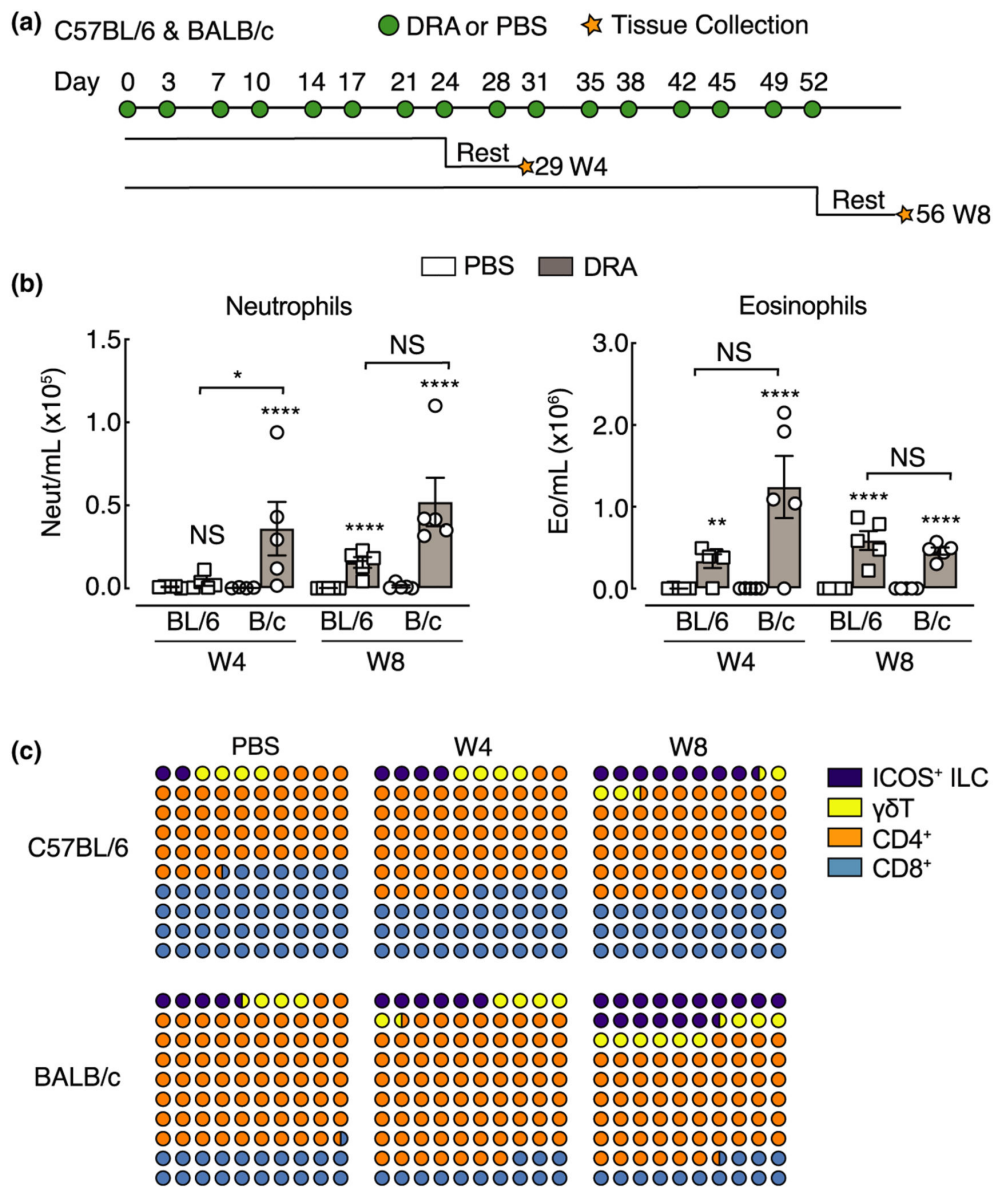
Chronic exposure to DRA allergens induces neutrophil and eosinophil airway inflammation. **(a)** Schematic showing an allergic airway inflammation model highlighting the timing of DRA or PBS intranasal administration into C57BL/6 or BALB/c mice. Cells were collected for flow cytometry analysis 5 days after the last administration of PBS or DRA (rest) at either 4 or 8 weeks. **(b)** Numbers of neutrophils and eosinophils in the BAL of C57BL/6 or BALB/c mice administered PBS or DRA for 4 or 8 weeks. **(c)** Plot showing the average proportions of different T cells and ILCs in the lungs of C57BL/6 or BALB/c mice administered PBS or DRA for 4 or 8 weeks. Data are representative of two experiments. Data are plotted as mean ± s.e.m. with points representing individual animals **(b)**. Data in **b** were analyzed by ANOVA with Tukey’s multiple comparison test with significance level showing comparisons between either PBS animals within each strain and each time point or C57BL/6 and BALB/c mice as indicated on the graph. *P < 0.05, **P < 0.01, ****P < 0.0001. BAL, bronchoalveolar lavage; DRA, house dust mite, ragweed and *Aspergillus fumigatus*; ILCs, innate lymphoid cells; NS, not significant; PBS, phosphate-buffered saline.

**Figure 2:**
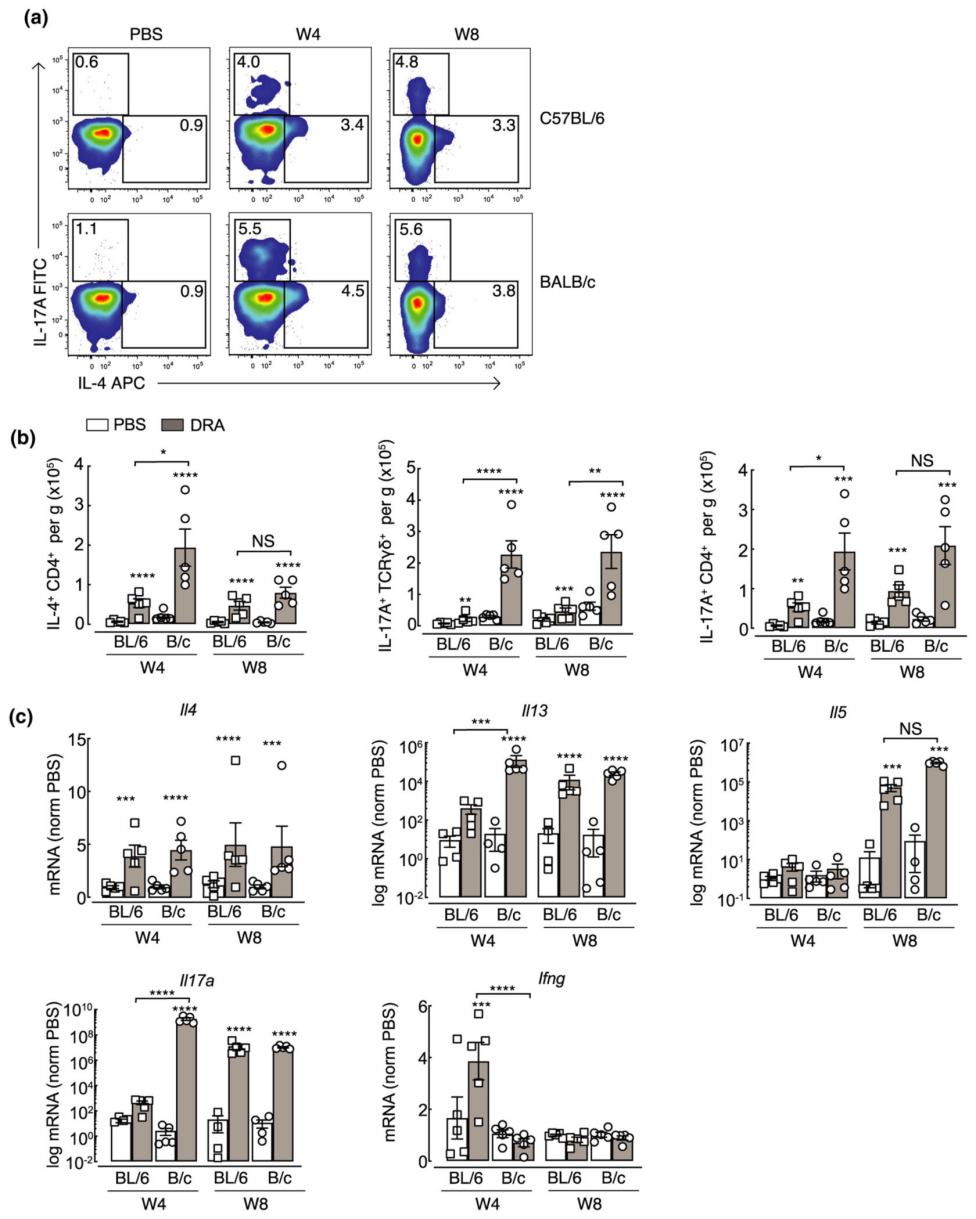
Chronic exposure to DRA allergens induces mixed Th2/Th17 airway inflammation. **(a)** Whole lung single-cell suspensions were stained for flow cytometry. Live, single TCRɣδ^–^TCRβ^+^ CD8^–^CD4^+^ cells were gated on and representative intracellular staining plots of IL-4^+^ and IL-17A^+^ CD4^+^ T cells in the lungs of C57BL/6 or BALB/c mice administered PBS or DRA intranasally twice a week for up to 8 weeks. Cells were analyzed by flow cytometry 5 days after the last instillation of allergen. Single-cell lung suspensions were stimulated with phorbol myristate acetate/ionomycin prior to analysis by flow cytometry. Numbers indicate the percentage of cytokine-positive CD4^+^ T cells within each gate. **(b)** Absolute numbers of IL-17A^+^ TCRɣδ^+^ or IL-17A^+^ or IL-4^+^ CD4^+^ T cells in the lungs of mice as in **a. (c)** mRNA expression of *Il4, Il13, Il5, Il17a* and *Ifng* in whole lungs of mice treated as in **a**. mRNAs were normalized to levels found in PBS C57BL/6 or BALB/c mice at each time point and are relative to geometric mean of housekeeping genes *Gapdh, Rpl13a* and *Rn45s*. Data are representative of two experiments. Data are plotted as mean ± s.e.m. with points representing individual animals. Data were analyzed by ANOVA with Tukey’s multiple comparison test with significance level showing comparisons between either PBS animals within each strain and each time point or C57BL/6 and BALB/c mice as indicated on the graph. *P < 0.05, **P < 0.01, ***P < 0.001, ****P < 0.0001. DRA, house dust mite, ragweed and *Aspergillus fumigatus*; IL, interleukin; mRNA, messenger RNA; NS, not significant; PBS, phosphate-buffered saline; TCR, T-cell receptor; Th2, T helper type 2; Th17, T helper type 17.

Aspects of allergen-induced airway inflammation have been extensively studied in mouse models, largely in the context of acute T helper type 2 (Th2)-mediated immune responses.^29,30^ Here, using a model of mixed type 2 and type 17 inflammation, we characterized immune cell dynamics in two common in-bred mouse strains, C57BL/6 and BALB/c, that are known to respond differently in models of airway inflammation and hyper-responsiveness.^26,31,32^ Mice were exposed to a multiallergen cocktail [house dust mite, ragweed and *Aspergillus fumigatus* (DRA)] for 4 or 8 weeks (Figure 1a) and inflammation assessed. All mice exposed to DRA had a mixed neutrophilic/eosinophilic inflammation, although neutrophilia was not evident in C57BL/6 mice until week 8 (Figure 1b). Neutrophils were still present in the bronchoalveolar lavage (BAL) 5 days after the last allergen administration (Figure 1a, b), even though their numbers were considerably lower compared with eosinophils (Figure 1b). Analysis of T lymphocytes and related populations in the lungs revealed relatively similar proportions of allergen-induced immune cell accumulation between mouse strains, with gradual increases in ICOS^+^ innate lymphoid cells (ILCs; Figure 1c). BALB/c mice generally had a greater ratio of CD4^+^ to CD8^+^ T cells compared with C57BL/6 mice, reflected in higher total lung CD4^+^ T-cell numbers (Supplementary figure 1), but this ratio did not change as a result of allergen exposure (Figure 1c). In addition, innate populations of γδT cells and ICOS^+^ ILCs were more predominant in BALB/c mice compared with C57BL/6 mice with clear increases in cell numbers in BALB/c mice following allergen exposure at either week 4 or 8 (Figure 1c and Supplementary figure 1). Despite the increased innate lymphoid cell numbers (Supplementary figure 1), CD4^+^ T cells and γδT cells appeared to be the major cytokine-producing lymphocyte populations in the lungs of all allergic mice. Exposure to DRA resulted in both IL-17A and type 2 inflammatory responses in the lung, with an increased proportion of IL-4 and IL-17A expressing CD4^+^ T cells in both C57BL/6 and BALB/c mice (Figure 2a). Interestingly, the numbers of IL-4^+^ CD4^+^ T cells were reduced from week 4 to week 8 in BALB/c mice (Figure 2b), also corresponding to a reduction in eosinophils (Figure 1b). Nonetheless, there were enhanced numbers of IL-17A^+^ TCRɣδ^+^ T cells and IL-17A^+^ CD4^+^ T as well as IL-4^+^ CD4^+^ T cells on week 4 in BALB/c compared with C57BL/6 mice (Figure 2b). Of note, TCRɣ δ^+^ and CD4^+^ T cells contributed equally to the pool of IL-17A^+^ cells in allergic BALB/c mice, whereas CD4^+^ T cells were the main IL-17A^+^ population in C57BL/6 mice (Figure 2b). Expression of key cytokines in whole lung RNA also revealed an increase in type 2 genes (*Il4, Il5, Il13*) and *Il17a* upon DRA treatment with exaggerated *Il13* and *Il17a* expression at week 4 in allergic BALB/c compared with C57BL/6 mice (Figure 2c). While we saw no evidence of increased IFNγ^+^ T cells or ILCs (data not shown) in the lungs of mice following allergen administration, there was a transient increase in *Ifng* expression in the lungs of C57BL/6 but not BALB/c mice (Figure 2c). Overall, although both mouse strains developed allergen-induced airway inflammation, the degree of inflammation and eosinophilic and neutrophilic responses were higher in BALB/c compared with C57BL/6 mice.

### Chitinase-like proteins are abundantly expressed in the lungs during type 2 and type 17 allergic airway inflammation

**Figure 3:**
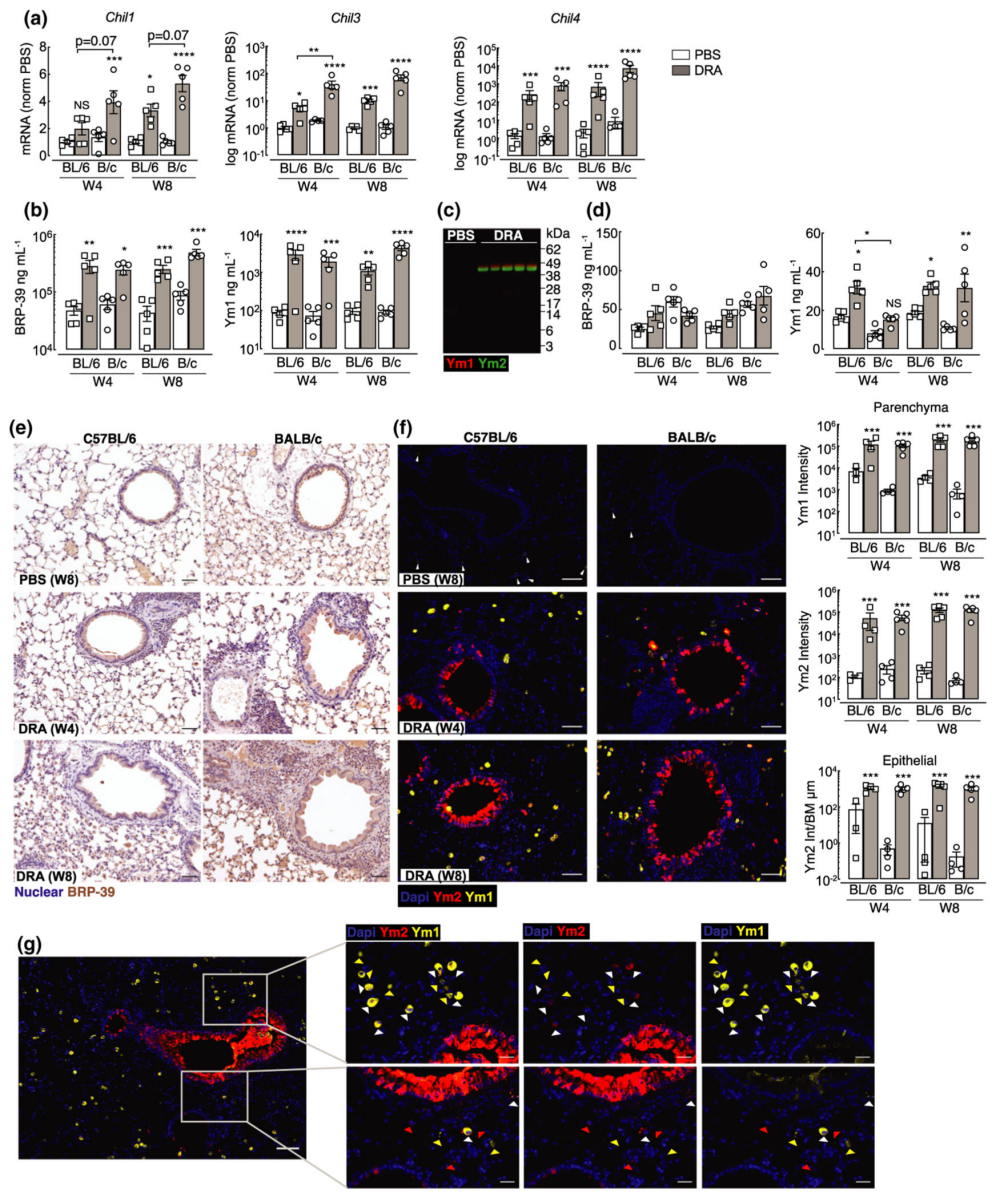
Chitinase-like proteins are abundantly expressed during chronic allergic airway inflammation. **(a)** mRNA expression of CLPs *Chil1, Chil3* and *Chil4* in whole lungs of C57BL/6 or BALB/c mice exposed intranasally to PBS or DRA for 4 or 8 weeks. Lungs were collected 5 days after the last PBS/DRA administration. mRNAs were normalized to levels found in PBS C57BL/6 or BALB/c mice at each time point and are relative to the geometric mean of housekeeping genes *Gapdh, Rpl13a* and *Rn45s. Chil3* and *Chil4* are depicted as log mRNA levels. **(b)** Concentration of Ym1 and BRP-39 protein measured by ELISA in the BAL of C57BL/6 or BALB/c mice treated as in **a**. **(c)** Western blot analysis of Ym1 (red) and Ym2 (green) levels in the BAL from C57BL/6 mice administered PBS or DRA for 8 weeks, with BAL taken 5 days after the last DRA/PBS administration. **(d)** Concentration of Ym1 and BRP-39 protein measured by ELISA in serum of C57BL/6 or BALB/c mice treated as in **a**. **(e)** Microscopy images of immunohistochemical staining of BRP-39 (brown) in lung sections from C57BL/6 and BALB/c mice treated with either PBS for 8 weeks, or DRA for 4 or 8 weeks. Cell nuclei counterstained with hematoxylin (purple); scale bar 50 µm. **(f)** Microscopy images of lung sections of mice as in a stained with DNA-binding dye (DAPI; blue), Ym1 (yellow) and Ym2 (red). Scale bar; 50 µm. Images are representative of five mice per group. Quantification of antibody-positive staining intensity from stained sections. Ym1 and Ym2 intensity in lung parenchyma areas and Ym2 intensity in airway epithelial cells normalized to length of airway basement membrane. **(g)** Microscopy images of immunofluorescent staining for Ym1 (yellow) and Ym2 (red) in lung sections for mice as in **f**. Images show areas where costaining in airway epithelial or parenchyma cells is evident. Triangles superimposed onto images show Ym1^+^Ym2^–^ cells (yellow), Ym1^–^Ym2^+^ cells (red) or Ym1^+^Ym2^+^ cells (white). Center image scale bar, 100 µm; outer images scale bar, 50 µm. Datapoints depict individual animals with bars representing mean and s.e.m. (**a, b, d, f**). Data are representative of two experiments. Data were analyzed by ANOVA with Tukey’s multiple comparison test with significance level showing comparisons between either PBS animals within each strain and each time point or C57BL/6 and BALB/c mice as indicated on the graph. *P < 0.05, **P < 0.01, ***P < 0.001, ****P < 0.0001. BAL, bronchoalveolar lavage; CLP, chitinase-like proteins; DAPI, 4′,6-diamidino-2-phenylindole; DRA, house dust mite, ragweed and *Aspergillus fumigatus*; mRNA, messenger RNA; NS, not significant; PBS, phosphate-buffered saline.

Chitinase-like proteins are molecules strongly associated with severe asthma, neutrophilia and IL-17A.^33–36^ Following exposure to DRA allergen, mRNA expression of murine chitinase-like genes *Chil1, Chil3* and *Chil4* were upregulated in both BALB/c and C57BL/6 mice compared with PBS controls (Figure 3a). However, *Chil1* mRNA expression was less in C57BL/6 compared with BALB/c mice, although this did not reach statistical significance in this data set (*P* = 0.07 for DRA C57BL/6 *versus* DRA BALB/c mice at week 4 or 8). In addition, no significant increase in *Chil1* mRNA was detected in whole lung tissue of C57BL/6 mice after 4 weeks of allergen exposure as compared with PBS controls (Figure 3a) despite significant increases in secreted BRP-39 protein levels in the BAL (Figure 3b). *Chil3* and *Chil4* were significantly increased in both mouse strains at week 4, and expression levels did not change upon further allergen exposure (Figure 3a), findings that were supported by measurement of Ym1 secreted protein in the BAL (Figure 3b). As there were no commercially available reagents to measure Ym2 protein levels, we developed a Ym2-specific antibody to examine Ym2 expression in the lungs (Supplementary figure 2). By western blot, neither Ym1 nor Ym2 was detected in mice administered PBS, but both Ym1 and Ym2 greatly increased following allergen exposure (Figure 3c). Chitinase-like proteins can be readily detected in the serum, and serum levels of YKL-40 in humans have been proposed as a biomarker for disease severity and are associated with reduced lung function in several pulmonary pathologies.^37–39^ Although BRP-39 is a genetic ortholog of YKL-40, the level of BRP-39 in the blood was not significantly altered following allergic inflammation in this model (Figure 3d). However, increased serum Ym1 was detectable in allergic mice of both strains (Figure 3d). To determine whether localization of the three chitinase-like proteins differed between strains of mice following allergen exposure, we examined immunostained lung sections. BRP-39 was already expressed in macrophages and epithelial cells in the steady state, but the intensity and number of positive cells increased further following allergen administration and the increase was particularly evident in BALB/c mice (Figure 3e). Corresponding to secreted levels in the BAL (Figure 3c), expression of Ym2 was absent in the lungs of PBS mice, while numerous Ym1^+^ cells, such as alveolar macrophages, could be detected (Figure 3f).^40^ The expression of Ym1 and Ym2 dramatically increased in the lungs of allergic BALB/c or C57BL/6 mice and the level of expression reached its maximum expression at 4 weeks post-DRA treatment (Figure 3f). Interestingly, Ym1 and Ym2 appear to have a fairly distinct expression pattern in the lung, with Ym1 largely restricted to myeloid cells and Ym2 largely expressed by epithelial cells, and there were very few cells that costained for Ym1 and Ym2 (Figure 3f, g). Overall, we observed modest increases in BRP-39 levels in allergic animals, but strongly enhanced Ym1 and Ym2 expression in the lungs of both allergic C57BL/6 and BALB/c mice. For the first time, we show distinct expression of Ym2 in the lungs compared with Ym1, despite their protein sequence being about 96% homologous.

### Allergen-induced immune pathways are fundamentally different between C57BL/6 and BALB/c mouse strains

**Figure 4:**
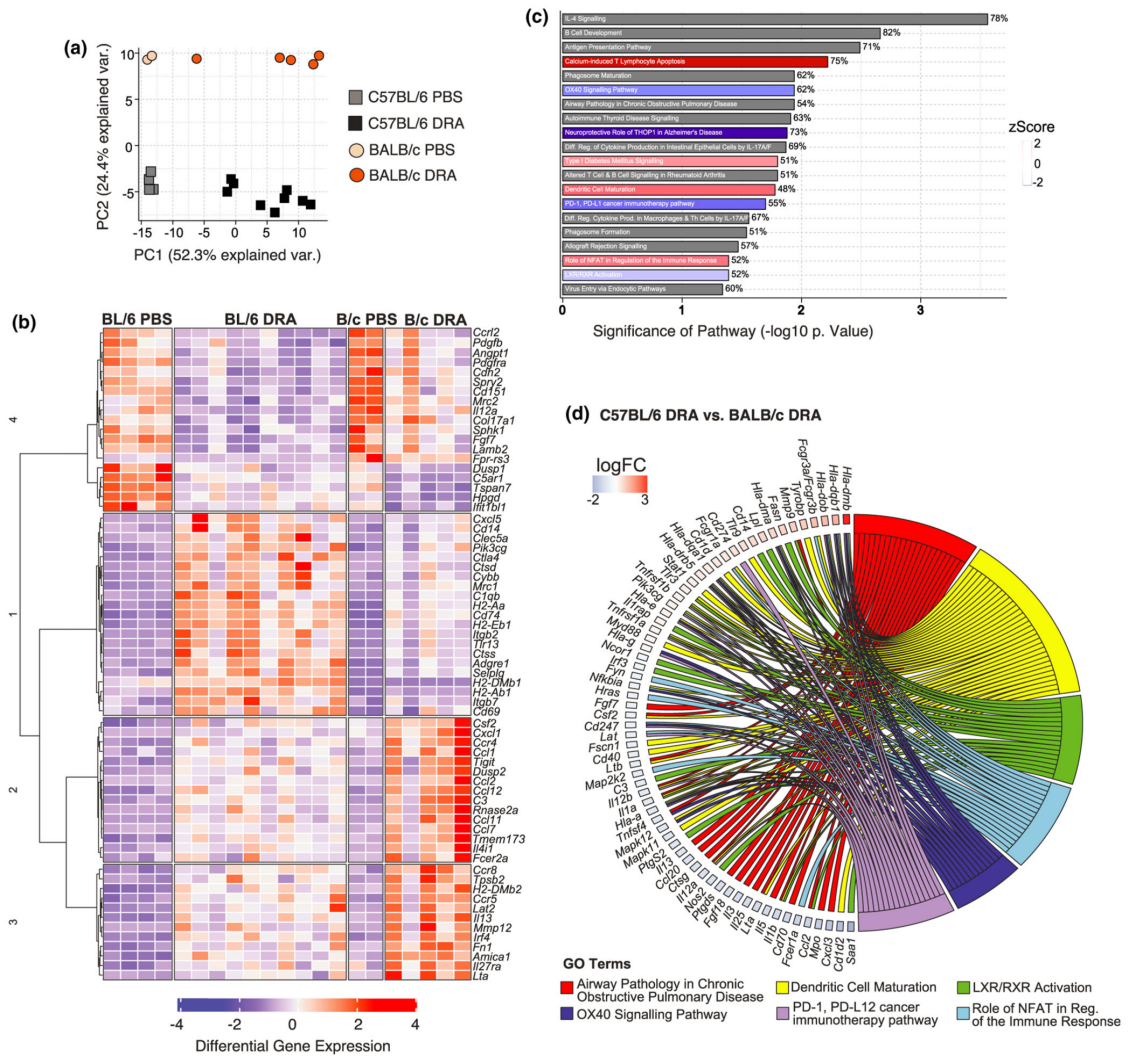
C57BL/6 and BALB/c allergic mice have fundamental differences in immune gene signatures. Whole lung RNAs from C57BL/6 and BALB/c mice administered with either PBS or DRA for 8 weeks were analyzed using NanoString Myeloid Panel version 2. **(a)** PCA of expressed genes from C57BL/6 and BALB/c. **(b)** Unsupervised, hierarchically clustered heatmap of genes that were significantly regulated in C57BL/6 and BALB/c allergic mice compared with PBS mice, but also differentially regulated between the treated strains. **(c)** Differentially expressed genes were visualized with the Ingenuity Pathway Analysis tool and the top 20 canonical pathways are shown for C57BL/6 *versus* BALB/c mice. Red or blue indicates pathways upregulated or downregulated, respectively, in C57BL/6 compared with BALB/c allergic mice. Gray indicates pathways that are significantly regulated but not in a particular direction. The percentage at the end of the bar equates to the number of molecules detected compared with the total number of molecules within the canonical pathway. **(d)** Chord diagram shows specific genes upregulated or downregulated (color indicating log fold change) within the GO term that were found to be significantly regulated in C57BL/6 allergic mice compared with BALB/c allergic mice. Transcriptomic analysis was performed on one experiment that was representative of two individual experiments. DRA, house dust mite, ragweed and *Aspergillus fumigatus*; GO, gene ontology; LXR, liver X receptor; NFAT, nuclear factor of activated T cells; PCA, principal component analysis; PBS, phosphate-buffered saline; RXR, retinoid X receptor.

Both C57BL/6 and BALB/c mice developed neutrophilic and eosinophilic airway inflammation in response to chronic allergen administration, despite a greater magnitude of both type 2 and IL-17A cytokine responses in BALB/c mice. Therefore, to more broadly characterize the differences in immune response between mouse strains, we performed differential gene expression analysis of whole lung RNA after 8 weeks of allergen or PBS administration using the NanoString nCounter Myeloid Innate Immunity Panel (NanoString, Amersham, UK). Principal component analysis demonstrated a clear separation in gene signatures not only from exposure to DRA *versus* PBS, principal component 1, but also between mouse strains, explained by principal component 2 (Figure 4a). Investigation of the genes that were significantly altered in the DRA model showed that numerous genes were induced (e.g. type 2 effector molecules *Retnla* and *Arg1*) or inhibited (e.g. basement membrane collagens, *Col4a1, Col4a2*) to an equivalent degree in both strains (Supplementary figure 3a). Hierarchical clustering also separated a considerable number of genes that were regulated in the same way between the strains, but to a much higher degree in one mouse strain over the other (Figure 4b) or expression of genes that were fundamentally different between strains (Supplementary figure 3b). As predicted from the allergic cytokine responses and alterations in immune cell infiltration into the lung (Figures 1 and 2), it was not surprising that type 2-related genes such as *Il13, Fcer2a, Csf2, Ccl2, Ccl11* were more highly upregulated in whole lung tissue from BALB/c compared with C57BL/6 mice (Figure 4b). However, interesting factors known to play an important role in leukocyte adhesion (*Itgb2, Itgb7, Selplg*) were upregulated in allergic C57BL/6 mice but not in BALB/c mice (Figure 4b), despite an apparent slower rate of inflammatory cell accumulation in C57BL/6 compared with BALB/c mice (Figure 1b and Supplementary figure 1).

Analysis of common properties within a signaling pathway (canonical pathway) showed enrichment of various pathways in C57BL/6 compared with BALB/c mice (Figure 4c). Pathways including “IL-4 signaling” and “airway pathology in chronic obstructive pulmonary disease” were significantly different between mouse strains (Figure 4c). Whether these pathways were activated or inhibited in C57BL/6 compared with BALB/c mice could not be clearly defined by the analysis (as denoted by the gray bar; Figure 4c). However, the specific genes that contributed to the z scores (Figure 4c) were also examined (Figure 4d). For example, a downregulation of both type 2 cytokines (*Il5* and *Il13*) and the type-2-inducing cytokine *Il25* in addition to reduced expression of proinflammatory cytokines *Il1a, Il1b, Il12a* and *Il12b* indicates that genes characteristic of the “airway pathology in chronic obstructive pulmonary disease” pathway (Figure 4d) were reduced in allergic C57BL/6 mice relative to BALB/c mice. Interestingly, both “OX40 signaling” and “PD-1, PD-L1 signaling,” pathways involved in maintenance and regulation of T-cell responses, respectively, were downregulated in allergic C57BL/6 compared with BALB/c mice (Figure 4d). These and other changes in canonical pathways involved in dendritic cell T-cell stimulation possibly explain reduced cytokine production in C57BL/6 mice (Figure 2). In addition, liver X receptor/retinoid X receptor activation, which maintains cholesterol homeostasis but is also known to be antifibrotic and anti-inflammatory,^41^ was downregulated in C57BL/6 mice (Figure 4c, d). Overall, analysis of gene regulation at chronic allergic inflammatory time points revealed differences in gene signatures between mouse strains that may explain reduced immune responses in C57BL/6 compared with BALB/c mice.

### Airway remodeling develops in both C57BL/6 and BALB/c mice despite different allergic inflammation dynamics and immune signatures

**Figure 5:**
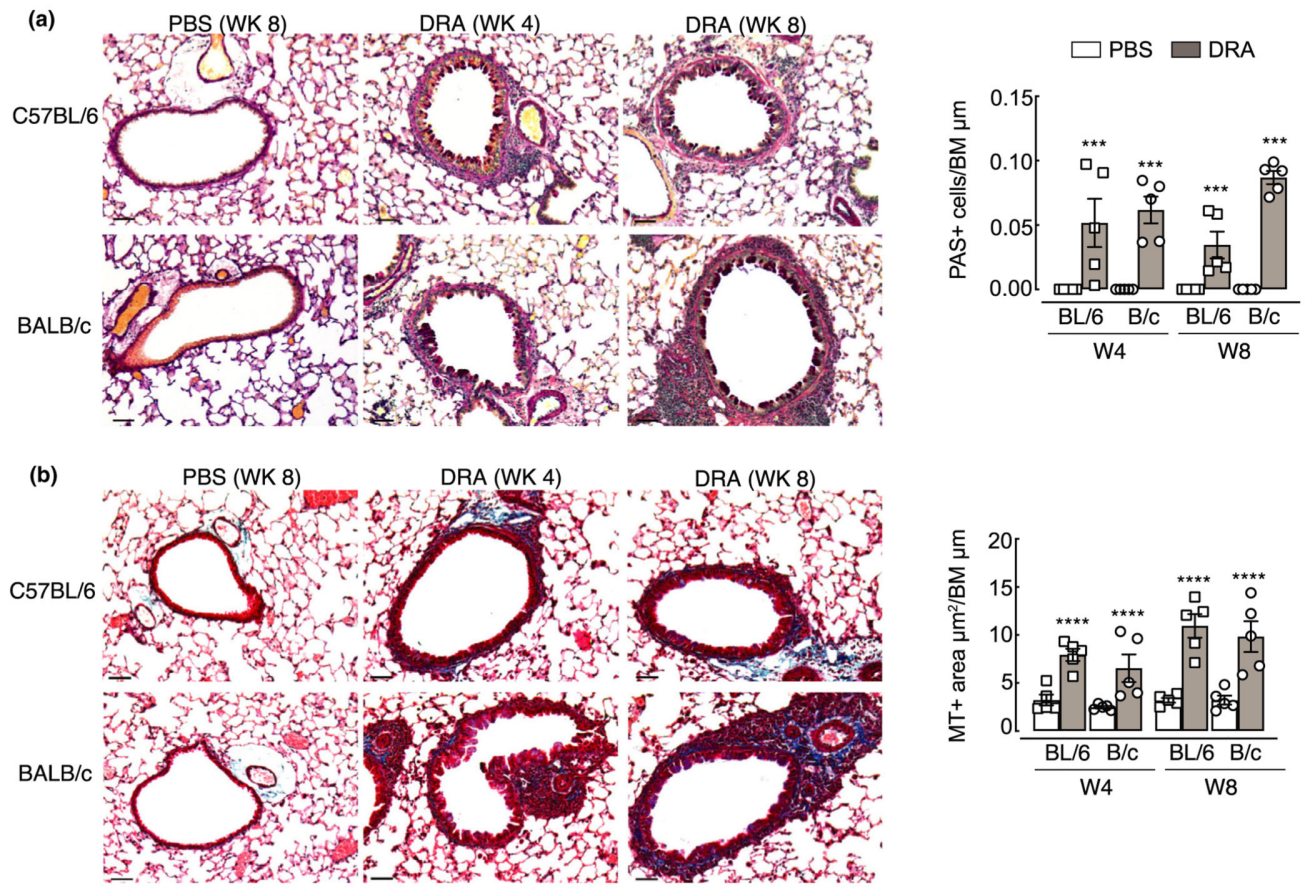
Goblet cell numbers and total collagen increase around the airways following exposure to DRA allergens. C57BL/6 or BALB/c mice were intranasally administered PBS or DRA twice a week for up to 8 weeks, and lungs were collected for histological analysis 5 days after the last PBS or DRA administration at weeks 4 and 8. **(a)** Microscopy images of lung sections stained for PAS. Airways show PAS^+^ cells (purple) within the epithelium. Graph shows quantification of numbers of PAS^+^ cells per length of basement membrane. **(b)** Microscopy images of lung sections stained for Masson’s trichrome (MT) from C57BL/6 or BALB/c mice. Airways show accumulation of collagen (blue) below the basement membrane. Graph shows quantification of the area of MT-positive staining around the airways normalized to basement membrane length. All images are representative of five mice; scale bar equals 50 µm. Datapoints depict individual animals with bars representing mean and s.e.m. Data are representative of two experiments and were analyzed by ANOVA with Tukey’s multiple comparison test and significance level shown relative to PBS animals within each strain and each time point. ***P < 0.001, ****P < 0.0001. BM, basement membrane; DRA, house dust mite, ragweed and *Aspergillus fumigatus*; PAS, periodic acid Schiff; PBS, phosphate-buffered saline.

**Figure 6:**
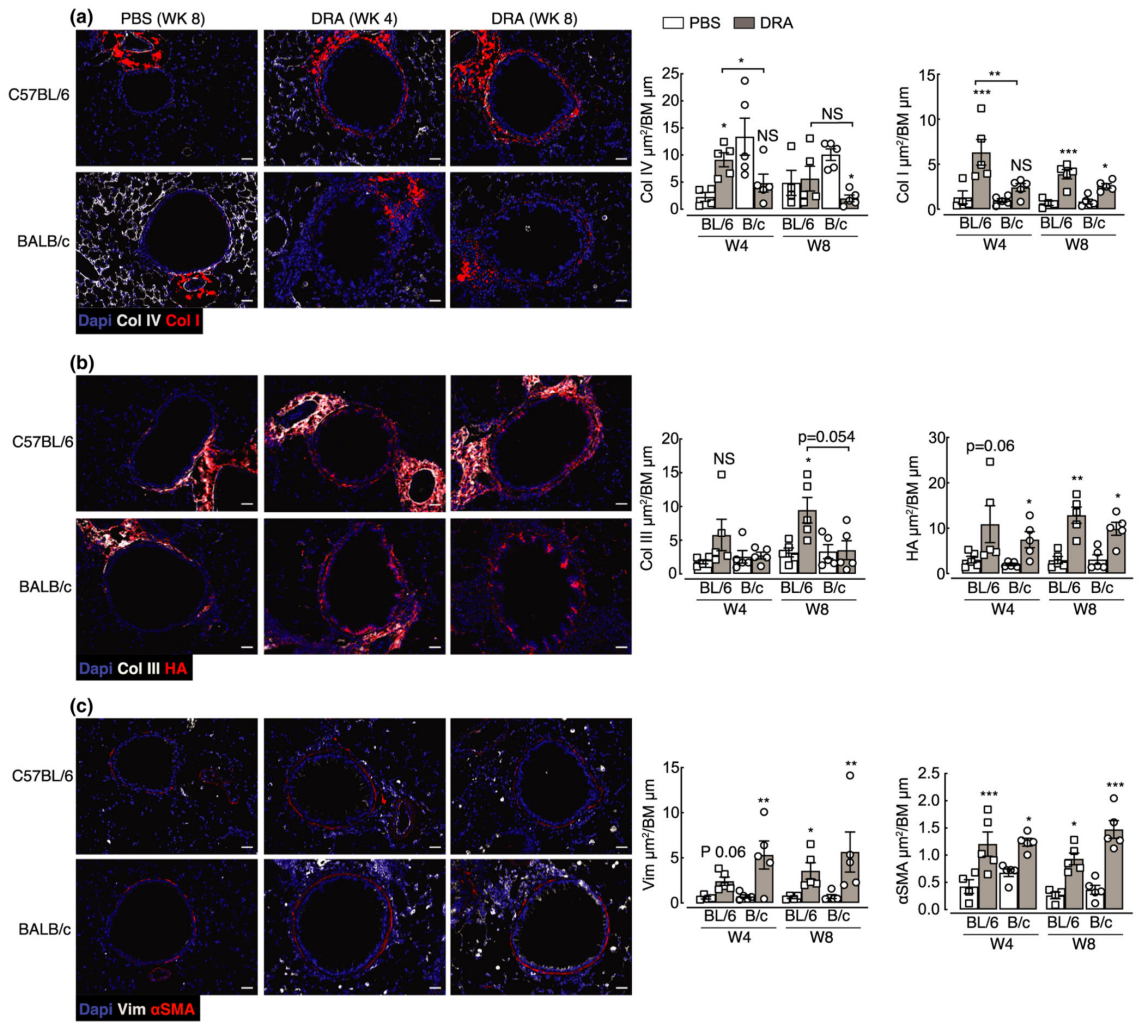
Changes to the ECM and muscle mass around the airway occur following exposure to DRA allergens. C57BL/6 or BALB/c mice were intranasally administered PBS or DRA twice a week for up to 8 weeks, and lungs were collected for immunostaining 5 days after the last PBS or DRA administration at weeks 4 and 8. **(a–c)** Microscopy images of lung sections from C57BL/6 or BALB/c mice stained with DNA-binding dye (DAPI; blue); **(a)** collagen IV (white) and collagen I (red); **(b)** collagen III (white) and HA-binding protein (red); **(c)** vimentin (Vim; white) and alpha smooth muscle actin (αSMA; red). Scale bar, 30 µm. Images are representative of five mice. Antibody-positive staining area was quantified around the airway and normalized to basement membrane length and values are depicted in **a–c**. Datapoints depict individual animals with bars representing mean and s.e.m. Data are representative of two experiments and were analyzed by ANOVA with Tukey’s multiple comparison test and significance level showing comparisons between either PBS animals within each strain and each time point or C57BL/6 and BALB/c mice as indicated on the graph. *P < 0.05, **P < 0.01, ***P < 0.001. Coll, collagen; BM, basement membrane; DAPI, 4′,6-diamidino-2-phenylindole; DRA, house dust mite, ragweed and *Aspergillus fumigatus*; ECM, extracellular matrix; HA, hyaluronan; PBS, phosphate-buffered saline.

The relationship between inflammation and airway remodeling in asthma is still controversial (reviewed by Saglani & Lloyd,^42^ Boulet,^43^ Guida & Riccio^44^). Some features of remodeling may occur in parallel or even prior to excessive inflammation,^18–20^ although this is difficult to test in the clinical setting. Considering different immune cell dynamics between BALB/c and C57BL/6 mice (Figures 1–4), we sought to determine whether features of airway remodeling also varied between mouse strains. Goblet cell hyperplasia is a key feature of remodeling in asthma and contributes to excessive airway mucus secretion. Equivalent increases in periodic acid Schiff-positive cells, indicative of goblet cells, were observed in both C57BL/6 and BALB/c mice at weeks 4 and 8 (Figure 5a). Airway remodeling in asthmatic patients is also characterized by thickening of the basement membrane and deposition of subepithelial extracellular matrix proteins. Following DRA allergen exposure, increased collagen deposition, measured by Masson’s trichrome stain, was also evident around the airways of both mouse strains (Figure 5b). Specific immunostaining for components of the extracellular matrix (ECM; Figure 6a, c) previously described to be regulated in asthma^45–48^ supported increases in total airway collagen following allergen exposure (Figure 5b). However, fundamental differences in collagen expression between mouse strains were also evident (Figure 6a, b). Basement membrane protein collagen IV alpha 1 was highly expressed in the steady state around the airways and alveoli of BALB/c mice compared with C57BL/6 (Figure 6a). Upon allergen administration, collagen IV alpha 1 expression decreased over time in BALB/c mice, whereas its levels transiently increased in C57BL/6 mice. In addition, a greater and more rapid increase in airway collagen I in allergic C57BL/6 compared with BALB/c mice was observed (Figure 6a) and similarly, accumulation of airway collagen III was significantly increased only in allergic C57BL/6 mice (Figure 6b). By contrast, expression of a major glycosaminoglycan component of the ECM, hyaluronan, was increased in response to allergen exposure independently of mouse strain (Figure 6b). Changes to collagen composition around the airways of allergic mice were accompanied by an increase in the number of vimentin-positive cells (Figure 6c), potentially indicating an increase in matrix-secreting fibroblasts.^49^ In addition, airway muscle mass was examined which revealed increases in mice administered DRA allergens regardless of mouse strain (Figure 6c). Together, these results demonstrate that features of remodeling such as goblet cell hyperplasia, increased smooth muscle mass and ECM changes occur in both C57BL/6 and BALB/c mice. However, differences in deposition of specific collagen subtypes exist between mouse strains.

## Discussion

IL-17 and neutrophilia are often associated with severe asthma.^10^ Despite this, models of allergic airway inflammation still largely focus on studying the regulation of allergen-induced type 2 immune responses, utilizing BALB/c mice that generally show a strongly skewed type 2 inflammatory response.^26^ Here, we utilized a model of allergic airway inflammation in which neutrophilia and IL-17 are dominant features, with inflammation resistant to steroid intervention^28^ and AHR unaffected by neutralization of IL-5 or IL-13 cytokines.^27^ As expected, BALB/c mice developed rapid and prominent airway inflammation that was skewed toward type 2 responses, but also showed greater IL-17 production, particularly by γδ T cells. However, type 2 inflammation was reduced from week 4 to week 8, perhaps reflecting the emergence of a tolerogenic response to allergens in BALB/c mice.^25^ C57BL/6 mice still responded to allergens, but Th2 and IL-17A responses developed at a slower rate compared with BALB/c mice. Delayed type 2 cytokine expression is potentially explained by an early but transient spike in interferon-gamma (IFNγ) expression only observed in C57BL/6 mice. Interestingly, increased IL-17A expression in C57BL/6 mice between weeks 4 and 8 coincided with a reduction in IFNγ levels in allergic mice, which we have shown previously to be an important factor that allows the development of a pulmonary type 2 immune response.^50^ In addition, IL-10 derived from T cells has been shown to signal via alveolar macrophages, leading to suppression of IFNγ-induced airway epithelial disruption.^51^ No difference in expression of *Il10* between strains at chronic time points was observed after DRA administration in our study. However, temporal changes in IL-10 in C57BL/6 mice may contribute to suppression of IFNγ alongside IL-17A.

IL-13 production is known to be higher in BALB/c *versus* C57BL/6 mice,^26^ as also shown here, and is thought to account for increased AHR observed in BALB/c compared with the relatively hyporesponsive C57BL/6 mice.^22,52^ In fact, type 2 cytokine-producing cells, rather than eosinophilic inflammation, appear to be key for the maintenance of AHR in models of type 2 airway inflammation.^53,23^ In addition to enhanced type 2 cytokine production, pathway analysis suggested a reduced capacity (in C57BL/6 mice) for antigen-presenting cell-mediated activation of T cells via costimulatory molecules PD-1/PD-L1 and OX40/OX40L, despite enhanced “dendritic cell maturation” pathways from NanoString analysis in these mice. Different dendritic cell subsets can dictate the allergic immune response and targeting either dendritic cell activation or molecules involved in antigen presentation may be a fruitful approach to therapeutically target allergic asthma, and specifically different immune phenotypes of disease.^54,55^ PD-L1 is known to enhance AHR and Th2 cytokine production in allergic mice.^56^ Therefore, a reduction in PD-1–PD-L1 signaling, alongside reduced OX40 signaling, may explain the reduced Th2 response in C57BL/6 mice compared with BALB/c. Future research identifying specific dendritic cell phenotypes in both strains during allergic airway inflammation could prove useful for understanding pathways to target AHR and inflammation in asthma.

In this model of Th2/Th17 allergic airway inflammation, both BALB/c and C57BL/6 mice developed a similar degree of airway remodeling in response to allergen exposure. However, our study reveals intriguing differences in ECM composition between mouse strains, not only in response to allergens but also in the steady state. One could anticipate that changes in collagen composition, particularly those centered around the ratios of collagen I and III, could profoundly alter lung function and, along with varied immune responses, may contribute to well-reported differences in AHR measurement between mouse strains.^22,52^ Both collagen I and III play major roles in the structural integrity of tissues and are often coexpressed within the tissue, with collagen I contributing to tensile strength, and collagen III allowing tissue flexibility.^57^ Collagen III can modulate scar formation^58^ and during early active fibrosis levels of collagen III significantly increase.^59,60^ However, an increased ratio between collagen I and III occurs in infants diagnosed with chronic lung disease proceeding respiratory distress syndrome.^59^ In addition, a lack of collagen III can disturb the development of collagen fibril formation, resulting in functional failure of the organ.^61,62^ Here, allergic BALB/c mice appeared to have preferential increase in collagen I around the airways, with no significant changes to collagen III, although we cannot rule out expression of collagen III at time points earlier than week 4. A failure to induce collagen III during remodeling processes may in fact perturb lung function, perhaps contributing to increases in AHR often observed in BALB/c mice in response to allergen challenge.^22,26,52^ Differential dynamics in collagen IV alpha 1 expression between mouse strains is also intriguing, as collagen IV is crucial for barrier formation anchoring airway epithelial cells. The rapid loss of collagen IV alpha 1 in BALB/c mice may relate to significantly increased vimentin-positive cells around the airways at week 4, and potentially enhanced epithelial-to-mesenchymal transition leading to a more rapid remodeling response in BALB/c *versus* C57BL/6. Although both mouse strains feature a similar magnitude of allergen-induced remodeling, further analysis of the early dynamics, before week 4, and mechanisms leading to changes in the ECM in these two mouse strains may reveal important features of tissue remodeling in disease.

Remodeling is typically examined as a change in epithelial goblet hyperplasia, increased muscle mass and total collagen, but in this study of genetically distinct mouse strains, the term remodeling is much more complicated. Just as inflammation varies greatly between asthmatic cohorts, airway remodeling too may be considered an “umbrella” term, whereby different pathways are likely to be more or less important in different asthma phenotypes. A greater understanding of how ECM composition changes can alter lung mechanics/function, as well as how the differing ECM components can regulate immune cell recruitment and activation, will help us to understand the development of lung diseases such as asthma and whether approaches to target remodeling will prove useful in treating such chronic inflammatory diseases. Furthermore, it is interesting to speculate that different genetic strains of mice, rather than using different allergens or timings of allergen exposure, could prove more useful for modeling different trajectories of allergic asthma in people.

## Methods

### Animals and ethics

Wild-type (BALB/c or C57BL/6J on OlaHsd background) mice were obtained from a commercial supplier (Envigo, Hillcrest, UK). Experimental mice, all female, were between 7 and 10 weeks of age at the start of the experiment and were housed in individually ventilated cages maintained in groups of five animals in specific pathogen-free facilities at the University of Manchester. Mice were not randomized in cages, but each cage was randomly assigned to a treatment group. Sample size was calculated on the basis of the number of animals needed for detection of a 25% change in Masson’s trichrome-positive area around the airway in PBS *versus* allergic mice, with a *P*-value of <0.05, based on pilot experiments carried out with three mice per group. All animal experiments were performed in accordance with the UK Animals (Scientific Procedures) Act of 1986 under a Project License (70/8548) granted by the UK Home Office and approved by the University of Manchester Animal Welfare and Ethical Review Body. Euthanasia was performed by asphyxiation in a rising concentration of carbon dioxide.

### Model of allergic airway inflammation

Allergic airway inflammation was induced in mice in a similar manner as has been described previously.^27^ Allergen DRA cocktail comprising 5 µg house dust mite (*Dermatophagoides pteronyssinus*, 5450 EU, 69.23 mg per vial), 50 µg ragweed (*Ambrosia artemisiifolia*) and 5 µg *Aspergillus fumigatus* extracts (Greer Laboratories, Lenoir, NC, USA) was freshly prepared prior to each instillation. Mice were briefly anesthetized via inhalation of isoflurane, and 20 µL of DRA cocktail or PBS was administered via intranasal instilliation twice weekly for up to 8 weeks. Mice were rested for 5 days prior to performing BAL and collecting lung tissue.

### Isolation of cells from the BAL and lung tissue

Following exsanguination, BAL cells were obtained through cannulation of the trachea and washing (four times) the lungs with 0.4 mL PBS (Sigma Aldrich, St. Louis, MO, USA) containing 0.25% bovine serum albumin (Sigma Aldrich, St. Louis, MO, USA). Lungs were processed as previously described.^40^ In brief, a right lobe was removed and minced in 1 mL of Hank’s Balanced Salt Solution buffer containing 0.4 U mL^−1^ Liberase TL (Sigma Aldrich, St. Louis, MO, USA) and 80 U mL^−1^ DNase type I (Thermo Fisher Scientific, Waltham, MA, USA) for 25 min in a 37°C shaking incubator. Digestion was stopped with 2% fetal bovine serum (Thermo Fisher Scientific, Waltham, MA, USA) and 2 mM ethylenediaminetetraacetic acid prior to passing the suspension through a 70 µm cell strainer (Greiner Bio-One, Stonehouse, UK). Red blood cells were lysed (Sigma Aldrich, St. Louis, MO, USA) and total live BAL and lung cell counts assessed with ViaStain AOPI (Nexcelom Bioscience LLC, Lawrence, MA, USA) using a Cellometer Auto 2000 automated cell counter (Nexcelom Bioscience LLC, Lawrence, MA, USA).

### Flow cytometry

Equal cell numbers of each lung and BAL sample were stained for flow cytometry. Cells were washed with ice-cold PBS and stained with Live/Dead Aqua or Blue (Thermo Fisher Scientific, Waltham, MA, USA) for 10 min at room temperature. All samples were then incubated with Fc block (5 µg mL^−1^ CD16/CD32; BD Biosciences, San Diego, CA, USA) and 0.1% mouse serum in fluorescence-activated cell sorting buffer [PBS containing 0.5% bovine serum albumin and 2 mM ethylenediaminetetraacetic acid (Thermo Fisher Scientific, Waltham, MA, USA)] for 20 min before staining for specific surface markers with fluorescence-conjugated antibodies for 25 min at 4°C (Table 1) Following surface staining, cells were fixed with ICC fix (BioLegend, San Diego, CA, USA) and stored at 4°C until intracellular staining was performed or cells were acquired. For intracellular cytokine staining, cells were stimulated for 4 h at 37°C with phorbol myristate acetate (0.5 µg mL^–1^; Sigma Aldrich, St. Louis, MO, USA) and ionomycin (1 µg mL^–1^; Sigma Aldrich, St. Louis, MO, USA) and for 3 h at 37°C with Brefeldin A (10 µg mL^−1^; BioLegend, San Diego, CA, USA). Cell surfaces were stained and cells fixed as described above. All cells were permeabilized (eBioscience, San Diego, CA, USA) and then stained with antibodies for intracellular cytokines (Table 1). Cells were identified with the following markers: eosinophils F4/80^+^ CD11c^–^ CD11b^+^ SigF^+^; neutrophils Ly6G^+^ CD11b^+^ CD11c^–^; T cells TCRβ^+^ TCRɣδ^–^ and either CD4^+^ or CD8^+^; gamma delta T cells TCRβ^–^ TCRɣδ^+^ CD4^–^ CD8^–^; innate lymphoid cells (ILCs) CD90^+^ ICOS^+^ lineage^–^ (CD11b, Ly6G, Ly6C, CD11c, Ter119, NK1.1, B220, CD3). All samples were acquired with an FACS Canto II or 5-Laser Fortessa with BD FACS Diva software and analyzed with FlowJo software (versions 9 and 10; BD Biosciences, San Diego, CA, USA).

### RNA extraction and quantitative real-time-PCR

One right lung lobe was stored in RNAlater (Thermo Fisher Scientific, Waltham, MA, USA) prior to homogenization in QIAzol reagent (Qiagen, Hilden, Germany). RNA was prepared according to manufacturer’s instructions and stored at −70°C. Reverse transcription of 0.2–0.5 µg total RNA was performed using 50 U Tetro reverse transcriptase (Bioline, London, UK), 40 mM deoxynucleoside triphosphates (Promega, Madison, WI, USA), 0.5 µg primer for complementary DNA synthesis (Sigma Aldrich, St. Louis, MO, USA) and RNasin inhibitor (Promega, Madison, WI, USA). The transcripts for genes of interest were measured by real-time PCR with a LightCycler 480 II System (Roche, Basel, Switzerland) and a Brilliant III SYBR Green Master Mix (Agilent Technologies, Santa Clara, CA, USA) with specific primer pairs (Table 2). mRNA amplification was analyzed by second derivative maximum algorithm (LightCycler 480 software, version 1.5; Roche, Basel, Switzerland) and expression of the gene of interest was normalized to the geometric mean of three housekeeping genes, namely, *Rn45s, Rpl13a* and *Gapdh* (Table 2).

### Transcriptome profile and associated analysis

Quality of RNA extracted from lung tissue, as described above, was assessed with an Agilent 2200 TapeStation system prior to downstream analyses; samples with an RNA integrity number less than 5.5 were excluded. RNA concentration was determined using the Qubit TM RNA BR Assay Kit (Thermo Fisher Scientific, Waltham, MA, USA) and 100 ng RNA (per sample) run on a NanoString nCounter R FLEX system using the Myeloid Innate Immunity version 2 panel (XT-CSO-MMII2-12; note that the probes in this panel do not distinguish between *Chil3* and *Chil4*). Raw counts were uploaded onto nSolver version 4.0 using default settings. Non-normalized counts were exported, and subsequent analyses performed in R (version 3.6.3) using RStudio Version 1.2.5033 (2009-2019 RStudio, Inc, Boston, MA, USA). Positive controls were analyzed to ensure there was clear resolution at variable expression levels and negative controls were used to set a minimum detection threshold which was then applied to all samples. Data were normalized with edgeR using the upper quartile method and differential expression of genes was calculated by linear modeling accounting for sample quality weights with empirical Bayes smoothing using the limma-voom R packages.^63^ All genes expressed above the background threshold were used for principal component analysis. Genes with an absolute fold change of greater than 0.5 and a significance value of under 0.05 after correction for multiple comparisons using the Benjamini–Yekutieli method were defined as “differentially expressed” and taken forward for further analysis. Heatmaps were then generated from scaled normalized counts of DE genes using the ComplexHeatmaps R package. The networks and functional analyses of DE genes were generated with Ingenuity Pathway Analyzer (QIAGEN Inc., https://www.qiagenbio-informatics.com/products/ingenuity-pathway-analysis). Within the Ingenuity Pathway Analyzer software no tissue filtering was used and the user data set was defined as the reference. Pathway data were then imported into R for visualization using the ggplot package.

### Generating anti-Ym2 and determining antibody specificity

Anti-Ym2-specific antibodies were generated by Cambridge Research Biochemicals (Billingham, UK). The 9-amino acid sequence at the N terminal (CKASYRGEL) was used as the immunogen as it has almost no homology to the Ym1 sequence. Bacterial optimized expression plasmids for Ym1 and Ym2 were purchased from GenScript (Piscataway, NJ, USA). Plasmids were then transfected into competent *Escherichia coli* (BL21) by heat shock followed antibiotic selection against ampicillin (25 mg mL^−1^) and chloramphenicol (34 mg mL^−1^). To generate recombinant protein a small scraping of the stock sample was expanded in Luria-Bertani media including antibiotics until optical density reached between 0.6 and 1.0 at which point isopropyl β- d- 1-thiogalactopyranoside (0.1 M) was added to the cultures. The optical density was kept under 1.0 by diluting the culture with fresh media as required and left overnight. Thereafter, bacteria were pelleted and resuspended in loading buffer containing dithiothreitol (200 mM; Thermo Fisher Scientific, Waltham, MA, USA).

### Western blotting

Lysed Ym1- or Ym2-transfected *E. coli* cells and murine BAL were denatured in the presence of dithiothreitol (200 mM ; Thermo Fischer Scientific, Waltham, MA, USA) for 5 min at 95°C. Each sample (2–10 µL) or protein ladder (SeeBlue; Thermo Fisher Scientific, Waltham, MA, USA) was separated on Bis-Tris 4–12% gradient gel with 2-(*N*-morpholino) ethanesulfonic acid buffer (Thermo Fisher Scientific, Waltham, MA, USA) before transfer onto a polyvinylidene difluoride membrane. The membrane was washed in distilled water followed by incubation in blocking buffer (5% bovine serum albumin in 0.05% Tween-20 in PBS) for 60 min at room temperature on a rocking platform. Primary antibodies were used at 1:500 [rabbit anti-mouse Ym2, polyclonal (custom made) or goat anti-mouse Ym1 polyclonal; R&D Systems, Minneapolis, MN, USA] and incubated at room temperature overnight on a rocking platform. The membrane was then washed in 0.05% Tween-20 in PBS followed by secondary antibody detection (1:1000 anti-rabbit immunoglobulin G Cy3 and Streptavidin-Cy5; Thermo Fisher Scientific, Waltham, MA, USA) for 1 hour at room temperature. Membranes were imaged using a Gel Documentation System (Azure Biosystems, Cambridge Bioscience, Cambridge, UK).

### Histology and immunostaining

The left lung lobe was fixed perfused with 10% neutral buffered formalin (Sigma Aldrich, St. Louis, MO, USA) and incubated overnight before being transferred to 70% ethanol. Lungs were processed and embedded in paraffin, then sectioned (5 µm) and stained with Masson’s trichrome or periodic acid Schiff stains using standard protocols. Images were captured with a Leica microscope with a digital camera (DMC2900). For immunostaining with antibodies, lung sections were deparaffinized and heat-mediated antigen retrieval was performed using Tris-ethylenediaminetetraacetic acid buffer (10 mM Tris base, 1 mM ethylenediaminetetraacetic acid, 0.05% Tween-20 pH 8.0; incubation 20 min 95°C). Nonspecific protein was blocked with 2% normal donkey serum (Sigma Aldrich, St. Louis, MO, USA) in PBS containing 0.05% Tween-20 and 1% bovine serum albumin. If a biotin-labeled antibody or probe was used, avidin biotin blocking (Thermo Fisher Scientific) was performed prior to an overnight incubation at 4°C with primary antibodies (Table 3). Sections were washed in PBS before incubation with secondary antibodies (Table 3) for 1 h at room temperature followed by mounting with 4ʹ,6-diamidino-2-phenylindole containing Fluoromount (Southern Biotech, Birmingham, AL, USA). Images were captured with an EVOS FL imaging system (Thermo Fisher Scientific, Waltham, MA, USA). Analysis of images was performed using ImageJ software (version 2.09.0-rc69/1.52p) on sections where sample identification was blinded for the investigator and airways analyzed had to be intact and fit within a single microscope field of view 480 × 360 µm. Goblet cells were visualized on periodic acid Schiff-stained sections and numbers of periodic acid Schiff-positive cells were counted per airway and normalized to the length of the airway basement membrane. Total collagen area was calculated by measuring the area of Masson’s trichrome-positive stain (blue) around the airway and values were normalized to basement membrane length. For calculation of collagen, hyaluronan, vimentin and alpha-smooth muscle actin area, background autofluorescence was subtracted from all images based on pixel intensities of sections stained with secondary antibodies only. A region of interest was drawn parallel to the airway basement membrane at a distance of 50 µm. A threshold was applied to all images to incorporate positively stained pixels and area of positively stained pixels within the region of interest was calculated and normalized to the length of the basement membrane. All areas of the airway that contained a blood vessel was excluded from analysis to ensure measurements specifically related to airways and not vasculature. For all image analysis, between 5 and 15 airways were measured per mouse.

### Quantification of Ym1 and BRP-39

The levels of Ym1 and BRP-39 in the serum and BAL were measured by sandwich ELISA using DuoSet ELISA kits (R&D Systems, Minneapolis, MN, USA) as per manufacturer’s recommendation.

### Statistical analysis

Statistical analysis was performed using JMP Pro 12.2.0 for Mac OS X (SAS Institute Inc., Cary, NC, USA). Normal distribution of data was determined by optical examination of residuals, and each group was tested for unequal variances using Welch’s test. Differences between groups were determined by ANOVA followed by a Tukey–Kramer honest significant difference multiple comparison test or an unpaired two-tailed Student’s *t*-test as indicated in the figure captions. In some data sets, data were log-transformed to achieve normal distribution. Differences were considered statistically significant for *P*-values less than 0.05.

## Supplementary Material

Additional supporting information may be found online in the Supporting Information section at the end of the article.

Supplementary figures

## Figures and Tables

**Table 1:** Antibodies used for flow cytometry analysis

Antigen	Antibody clone	Isotype	Source
Ly6G	1A8	Rat IgG2a κ	BioLegend
CD11b	M1/70	Rat IgG2b κ	BioLegend
CD11c	N418	Armenian Hamster IgG	BioLegend
F4/80	BM8	Rat IgG2a κ	BioLegend
CD64	X54-5/7.1	Mouse IgG1 κ	BioLegend
SiglecF	E50-2440	Rat IgG2a κ	BD Biosciences
I-A/I-E	M5/144.15.2	Rat IgG2b κ	BioLegend
TCRβ	H57-597	Armenian Hamster IgG	eBioscience
TCRγδ	GL3	Armenian Hamster IgG	BioLegend
CD4	GK1.5	Rat IgG2b κ	BioLegend
CD8	53-6.7	Rat IgG2a κ	BioLegend
CD3	17A2	Rat IgG2b κ	BioLegend
B220	RA3-682	Rat IgG2a κ	BioLegend
Ter119	Ter-119	Rat IgG2b κ	BioLegend
NK1.1	PK136	Mouse IgG2a κ	BioLegend
ICOS	C398.4A	Armenian Hamster IgG	BioLegend
CD90.2	30-H12	Rat IgG2b κ	BioLegend
IL-4	11b11	Rat IgG1 κ	BioLegend
IL-13	eBio13A	Rat IgG1 κ	eBioscience
IL-17a	TC11-18H10.1	Rat IgG1 κ	BioLegend
IFNγ	XMG1.2	Rat IgG1 κ	BioLegend

**Table 2:** Sequences of primers for measurement of mRNA expression via quantitative real-time-PCR

Gene	Forward primer	Reverse primer
Il4	CCTGCTCTTCTTTCTCGAATG	CACATCCATCTCCGTGCAT
Il13	CCTCTGACCCTTAAGGAGCTTAT	CGTTGCACAGGGGAGTCT
Il5	ACATTGACCGCCAAAAAGAG	CACCATGGAGCAGCTCAG
Il17a	GCTCCAGAAGGCCCTCAGACT	CCAGCTTTCCCTCCGCATTGA
Ifng	GGAGGAACTGGCAAAAGGAT	TTCAAGACTTCAAAGAGTCTGAGG
Chil1	CCAGCCAGGCAGAGAGAAAC	GCCACCTTTCCTGCTGACA
Chil3	TCTGGTGAAGGAAATGCGTAAA	GCAGCCTTGGAATGTCTTTCTC
Chil4	TCTGGTGCAGGAAATGCGTAAA	GCAGCCTTGGAATGTGGTTCAAAG
Rn45s	GTAACCCGTTGAACCCCATT	CCATCCAATCGGTAGTAGCG
Rpl13a	CATGAGGTCGGGTGGAAGTA	GCCTGTTTCCGTAACCTCAA
Gapdh	ATGACATCAAGAAGGTGGTG	CATACCAGGAAATGAGCTTG

**Table 3:** Antibodies used for immuno-histological analysis

Antigen	Antibody clone	Dilution	Source (catalog number)
Ym1	Goat polyclonal—Biotinylated	1:100	R&D (BAF2446)
Ym2	Rabbit polyclonal	1:1000	Home-made (not applicable)
Collagen I	Goat polyclonal	1:200	Cambridge Bioscience (1310-01)
Collagen III (N-terminal)	Rabbit polyclonal	1:300	Proteintech (22734-1-AP)
Collagen IV alpha I	Rabbit polyclonal	1:200	Novus Biologicals (NB120-6586)
Hyaluronan binding protein	Biotinylated	1:100	Merck Millipore (385911)
α-Smooth muscle actin	Goat polyclonal	1:200	Novus Biologicals (NB-300-978)
Vimentin	Rabbit polyclonal	1:200	Abcam (ab45939)
BRP-39	Rabbit Polyclonal	1:100	Biorbyt (orb10365)
-	Streptavidin 557	1:800	R&D (NL999)
-	Streptavidin 637	1:400	R&D (NL998)
-	Donkey anti-rabbit IgG 557	1:200	R&D (NL004)
-	Donkey anti-rabbit IgG 637	1:200	R&D (NL005)
-	Donkey anti-goat IgG 557	1:200	R&D (NL001)
-	Donkey anti-goat IgG 637	1:200	R&D (NL002)

## References

[1] (2018). The Global Asthma Report, Auckland, New Zealand.

[2] Custovic A, Henderson J, Simpson A (2019). Does understanding endotypes translate to better asthma management options for all?. J Allergy Clin Immunol.

[3] Pavord ID, Beasley R, Agusti A (2018). After asthma: redefining airways diseases. Lancet.

[4] Kuruvilla ME, Lee FE-H, Lee GB (2019). Understanding asthma phenotypes, endotypes, and mechanisms of disease. Clin Rev Allergy Immunol.

[5] McGregor MC, Krings JG, Nair P, Castro M (2019). Role of biologics in asthma. Am J Respir Crit Care Med.

[6] Hansbro PM, Kaiko GE, Foster PS (2011). Cytokine/anti-cytokine therapy - novel treatments for asthma?. Br J Pharmacol.

[7] Pavord ID, Korn S, Howarth P (2012). Mepolizumab for severe eosinophilic asthma (DREAM): a multicentre, double-blind, placebo-controlled trial. Lancet.

[8] Haldar P, Brightling CE, Singapuri A (2014). Outcomes after cessation of mepolizumab therapy in severe eosinophilic asthma: a 12-month follow-up analysis. J Allergy Clin Immunol.

[9] Busse WW, Holgate S, Kerwin E (2013). Randomized, double-blind, placebo-controlled study of brodalumab, a human anti-IL-17 receptor monoclonal antibody, in moderate to severe asthma. Am J Respir Crit Care Med.

[10] Lindén A, Dahlén B (2014). Interleukin-17 cytokine signalling in patients with asthma. Eur Respir J.

[11] Beers MF, Morrisey EE (2011). The three R’s of lung health and disease: repair, remodeling, and regeneration. J Clin Invest.

[12] Hansbro PM, Kim RY, Starkey MR (2017). Mechanisms and treatments for severe, steroid-resistant allergic airway disease and asthma. Immunol Rev.

[13] Hoshino M, Takahashi M, Takai Y, Sim J, Aoike N (2001). Inhaled corticosteroids decrease vascularity of the bronchial mucosa in patients with asthma. Clin Exp Allergy.

[14] Laitinen A, Altraja A, Kämpe M, Linden M, Virtanen I, Laitinen LA (1997). Tenascin is increased in airway basement membrane of asthmatics and decreased by an inhaled steroid. Am J Respir Crit Care Med.

[15] Hoshino M, Takahashi M, Takai Y, Sim J (1999). Inhaled corticosteroids decrease subepithelial collagen deposition by modulation of the balance between matrix metalloproteinase-9 and tissue inhibitor of metalloproteinase-1 expression in asthma. J Allergy Clin Immunol.

[16] Flood-Page P, Menzies-Gow A, Phipps S (2003). Anti-IL-5 treatment reduces deposition of ECM proteins in the bronchial subepithelial basement membrane of mild atopic asthmatics. J Clin Invest.

[17] Zhao J, Lloyd CM, Noble A (2013). Th17 responses in chronic allergic airway inflammation abrogate regulatory T-cell-mediated tolerance and contribute to airway remodeling. Mucosal Immunol.

[18] Henschen M, Stocks J, Brookes I, Frey U (2006). New aspects of airway mechanics in pre-term infants. Eur Respir J.

[19] Saglani S, Payne DN, Zhu J (2007). Early detection of airway wall remodeling and eosinophilic inflammation in preschool wheezers. Am J Respir Crit Care Med.

[20] Lezmi G, Gosset P, Deschildre A (2015). Airway remodeling in preschool children with severe recurrent wheeze. Am J Respir Crit Care Med.

[21] Donohue JF, Ohar JA (2004). Effects of corticosteroids on lung function in asthma and chronic obstructive pulmonary disease. Proc Am Thorac Soc.

[22] Van Hove CL, Maes T, Cataldo DD (2009). Comparison of acute inflammatory and chronic structural asthma-like responses between C57BL/6 and BALB/c mice. Int Arch Allergy Immunol.

[23] Kearley J, Buckland KF, Mathie SA, Lloyd CM (2009). Resolution of allergic inflammation and airway hyperreactivity is dependent upon disruption of the T1/ST2-IL-33 pathway. Am J Respir Crit Care Med.

[24] Hirota JA, Ask K, Fritz D (2009). Role of STAT6 and SMAD2 in a model of chronic allergen exposure: a mouse strain comparison study. Clin Exp Allergy.

[25] Shinagawa K (2003). Mouse model of airway remodeling: strain differences. Am J Respir Crit Care Med.

[26] Gueders MM, Paulissen G, Crahay C (2009). Mouse models of asthma: a comparison between C57BL/6 and BALB/c strains regarding bronchial responsiveness, inflammation, and cytokine production. Inflamm Res.

[27] Goplen N, Karim MZ, Liang Q (2009). Combined sensitization of mice to extracts of dust mite, ragweed, and *Aspergillus* species breaks through tolerance and establishes chronic features of asthma. J Allergy Clin Immunol.

[28] Liu R, Bai J, Xu G (2013). Multi-allergen challenge stimulates steroid-resistant airway inflammation via NF-κB-mediated IL-8 expression. Inflamm.

[29] Voskamp AL, Kormelink TG, van Wijk RG (2020). Modulating local airway immune responses to treat allergic asthma: lessons from experimental models and human studies. Semin Immunopathol.

[30] Rosenberg HF, Druey KM (2018). Modeling asthma: Pitfalls, promises, and the road ahead. J Leukoc Biol.

[31] Sahu N, Morales JL, Fowell D, August A (2010). Modeling susceptibility versus resistance in allergic airway disease reveals regulation by Tec kinase Itk. PLoS One.

[32] De Vooght V, Vanoirbeek JAJ, Luyts K, Haenen S, Nemery B, Hoet PHM (2010). Choice of mouse strain influences the outcome in a mouse model of chemical-induced asthma. PLoS One.

[33] James AJ, Reinius LE, Verhoek M (2016). Increased YKL-40 and chitotriosidase in asthma and chronic obstructive pulmonary disease. Am J Respir Crit Care Med.

[34] Liu L, Zhang X, Liu Y (2019). Chitinase-like protein YKL-40 correlates with inflammatory phenotypes, anti-asthma responsiveness and future exacerbations. Respir Res.

[35] Ober C, Tan Z, Sun Y (2008). Effect of variation in *CHI3L1* on serum YKL-40 level, risk of asthma, and lung function. N Engl J Med.

[36] Konradsen JR, James A, Nordlund B (2013). The chitinase-like protein YKL-40: a possible biomarker of inflammation and airway remodeling in severe pediatric asthma. J Allergy Clin Immunol.

[37] Tong X, Wang D, Liu S (2018). The YKL-40 protein is a potential biomarker for COPD: a meta-analysis and systematic review. COPD.

[38] Chupp GL, Lee CG, Jarjour N (2007). A chitinase-like protein in the lung and circulation of patients with severe asthma. N Engl J Med.

[39] Furuhashi K, Suda T, Nakamura Y (2010). Increased expression of YKL-40, a chitinase-like protein, in serum and lung of patients with idiopathic pulmonary fibrosis. Respir Med.

[40] Sutherland TE, Ruckerl D, Logan N, Duncan S, Wynn TA, Allen JE (2018). Ym1 induces RELMα and rescues IL-4Rα deficiency in lung repair during nematode infection. PLoS Pathog.

[41] Smet M, Van Hoecke L, De Beuckelaer A (2016). Cholesterol-sensing liver X receptors stimulate Th2-driven allergic eosinophilic asthma in mice. Immun Inflamm Dis.

[42] Saglani S, Lloyd CM (2015). Novel concepts in airway inflammation and remodelling in asthma. Eur Respir J.

[43] Boulet L-P (2018). Airway remodeling in asthma: update on mechanisms and therapeutic approaches. Curr Opin Pulm Med.

[44] Guida G, Riccio AM (2019). Immune induction of airway remodeling. Semin Immunol.

[45] Chakir J, Shannon J, Molet S (2003). Airway remodeling-associated mediators in moderate to severe asthma: effect of steroids on TGF-β, IL-11, IL-17, and type I and type III collagen expression. J Allergy Clin Immunol.

[46] Benayoun L, Druilhe A, Dombret M-C, Aubier M, Pretolani M (2003). Airway structural alterations selectively associated with severe asthma. Am J Respir Crit Care Med.

[47] Johnson PRA, Burgess JK, Underwood PA (2004). Extracellular matrix proteins modulate asthmatic airway smooth muscle cell proliferation via an autocrine mechanism. J Allergy Clin Immunol.

[48] Lauer ME, Majors AK, Comhair S (2015). Hyaluronan and its heavy chain modification in asthma severity and experimental asthma exacerbation. Jl Biol Chem.

[49] Goodpaster T, Legesse-Miller A, Hameed MR, Aisner SC, Randolph-Habecker J, Coller HA (2008). An immunohistochemical method for identifying fibroblasts in formalin-fixed, paraffin-embedded tissue. J Histochem Cytochem.

[50] Ajendra J, Chenery AL, Parkinson JE (2020). IL-17A both initiates, via IFNγ suppression, and limits the pulmonary type-2 immune response to nematode infection. Mucosal Immunol.

[51] Branchett WJ, Stölting H, Oliver RA (2020). A T cell-myeloid IL-10 axis regulates pathogenic IFN-γ-dependent immunity in a mouse model of type 2-low asthma. J Allergy Clin Immunol.

[52] Kelada SNP, Wilson MS, Tavarez U (2011). Strain-dependent genomic factors affect allergen-induced airway hyperresponsiveness in mice. Am J Respir Cell Mol Biol.

[53] Walter DM, McIntire JJ, Berry G (2001). Critical role for IL-13 in the development of allergen-induced airway hyperreactivity. J Immunol.

[54] Cook PC, MacDonald AS (2016). Dendritic cells in lung immunopathology. Semin Immunopathol.

[55] Gaurav R, Agrawal DK (2013). Clinical view on the importance of dendritic cells in asthma. Expert Rev Clin Immunol.

[56] McAlees JW, Lajoie S, Dienger K (2015). Differential control of CD4^+^ T-cell subsets by the PD-1/PD-L1 axis in a mouse model of allergic asthma. Eur J Immunol.

[57] Silver FH, Freeman JW, Seehra GP (2003). Collagen self-assembly and the development of tendon mechanical properties. J Biomech.

[58] Xue M, Jackson CJ (2015). Extracellular matrix reorganization during wound healing and its impact on abnormal scarring. Adv Wound Care (New Rochelle).

[59] Shoemaker CT, Reiser KM, Goetzman BW, Last JA (1984). Elevated ratios of type I/III collagen in the lungs of chronically ventilated neonates with respiratory distress. Pediatr Res.

[60] Bateman ED, Turner-Warwick M, Adelmann-Grill BC (1981). Immunohistochemical study of collagen types in human foetal lung and fibrotic lung disease. Thorax.

[61] Asgari M, Latifi N, Heris HK, Vali H, Mongeau L (2017). *In vitro* fibrillogenesis of tropocollagen type III in collagen type I affects its relative fibrillar topology and mechanics. Sci Rep.

[62] Kuivaniemi H, Tromp G (2019). Type III collagen (*COL3A1*): Gene and protein structure, tissue distribution, and associated diseases. Gene.

[63] Ritchie ME, Phipson B, Wu D (2015). limma powers differential expression analyses for RNA-sequencing and microarray studies. Nucleic Acids Res.

